# Mortality Rates above Emergency Threshold in Population Affected by Conflict in North Kivu, Democratic Republic of Congo, July 2012–April 2013

**DOI:** 10.1371/journal.pntd.0003181

**Published:** 2014-09-18

**Authors:** Antonio Isidro Carrión Martín, Karla Bil, Papy Salumu, Dominique Baabo, Jatinder Singh, Corry Kik, Annick Lenglet

**Affiliations:** 1 Médecins Sans Frontières, Operational Centre Amsterdam, Amsterdam, The Netherlands; 2 Zone de Santé, Walikale, North Kivu, Democratic Republic of Congo; 3 Médecin Inspecteur Provinciale, Goma, North Kivu, Democratic Republic of Congo; 4 Médecins Sans Frontières, Operational Centre Amsterdam, Quartiers Les Volcans, Goma, North Kivu, Democratic Republic of Congo; University of Washington, United States of America

## Abstract

The area of Walikale in North Kivu, Democratic Republic of Congo, is intensely affected by conflict and population displacement. Médecins-Sans-Frontières (MSF) returned to provide primary healthcare in July 2012. To better understand the impact of the ongoing conflict and displacement on the population, a retrospective mortality survey was conducted in April 2013. A two-stage randomized cluster survey using 31 clusters of 21 households was conducted. Heads of households provided information on their household make-up, ownership of non-food items (NFIs), access to healthcare and information on deaths and occurrence of self-reported disease in the household during the recall period. The recall period was of 325 days (July 2012–April 2013). In total, 173 deaths were reported during the recall period. The crude mortality rate (CMR) was of 1.4/10,000 persons/day (CI95%: 1.2–1.7) and the under-five- mortality rate (U5MR) of 1.9/10,000 persons per day (CI95%: 1.3–2.5). The most frequently reported cause of death was fever/malaria 34.1% (CI95%: 25.4–42.9). Thirteen deaths were due to intentional violence. Over 70% of all households had been displaced at some time during the recall period. Out of households with someone sick in the last two weeks, 63.8% sought health care; the main reason not to seek health care was the lack of money (n = 134, 63.8%, CI95%: 52.2–75.4). Non Food Items (NFI) ownership was low: 69.0% (CI95%: 53.1–79.7) at least one 10 liter jerry can, 30.1% (CI95%: 24.3–36.5) of households with visible soap available and 1.6 bednets per household. The results from this survey in Walikale clearly illustrate the impact that ongoing conflict and displacement are having on the population in this part of DRC. The gravity of their health status was highlighted by a CMR that was well above the emergency threshold of 1 person/10,000/day and an U5MR that approaches the 2 children/10,000/day threshold for the recall period.

## Author Summary

The area of Walikale, in the eastern province of North Kivu, Democratic Republic of Congo has been experiencing ongoing conflict, violence and population displacement in recent years. Médecins Sans Frontières (MSF) has been working in this part of DRC since July 2012 and decided to document the impact of the conflict on the population by estimating the number of deaths that had occurred in this community between July 2012 and April 2013. Thirty-one villages (with 21 households each) were randomly selected; households were asked to report on the number of deaths, cases of disease, access to health care and ownership of items such as bednets and jerry cans. The overall mortality rate during the period was 1.4 deaths/10,000 person per day, which exceeded the emergency thresholds of 1/10,000 per day. In children under five years this was 1.9/10,000/day; close to the emergency thresholds of 2/10,000/day for this age group. Malaria and diarrhea were commonly reported causes of death and disease. Access to health care was limited due to financial constraints and ownership of bednets and jerrycans very low. The impact of the conflict in the health status of people in this part of DRC therefore appeared to be quite dramatic.

## Introduction

Since its independence in 1960, the Democratic Republic of the Congo (DRC) has continued to face a series of internal political and armed struggles [1] which contribute to an on-going humanitarian crisis in several parts of the country. According to the World Health Organisation (WHO), life expectancy at birth in DRC is 49 years and estimates from 2010 determine that the global under-five mortality rate (GU5MR) is 158 deaths per 1000 live births. In 2010, prevalence for fever (as an indicator of malaria) and diarrhoea in children under-five years of age was estimated at 27% and 18% respectively [2]. However, the true burden of malaria and other associated neglected diseases is difficult to estimate in DRC as surveillance for these diseases is practically absent [3].

**Figure 1:**
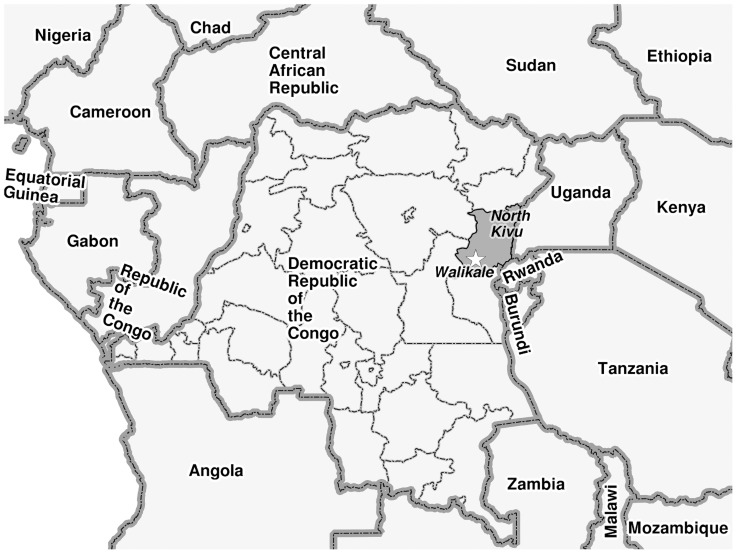
Map indicating Walikale in the province of North Kivu, Democratic Republic of Congo (DRC).

Crude mortality rates (CMRs), which are an indicator of the severity of a humanitarian situation within a population, are considered to be over the threshold when one death per 10,000 population is reported per day (for the under-five mortality rates, the threshold is 2 deaths/10,000 population/day) [4]. The province of North Kivu (see Figure 1) in eastern DRC, has been at the centre of conflict, displacement and instability in the country for almost two decades. The CMRs measured in the population in different parts of the province between 2002 and 2009 suggest that the acuity of the ongoing insecurity in the province is dynamic in time and geographical area. Between 2002 and 2006 estimated CMRs ranged between 0.97 and 1.2 deaths per 10000 population per day [5] [6]. In 2009, CMRs in Kabizo, Masisi and Kitchanga in North Kivu were estimated at 0.2, 0.5 and 0.7 deaths per 10000 population per day respectively [7].

In April 2012, the province witnessed sparked violence and widespread population displacement, the worst seen for many years. In the area of Walikale (see Figure 1), this resulted in more than 150,000 internally displaced persons moving to the town [8]. In July 2012 intense fighting also affected the town of Walikale itself. Médecins Sans Frontières (MSF) worked in Walikale between 2003 and 2008 and re-started activities in May 2012, focusing on vertical interventions for malaria diagnosis and treatment and strengthening the delivery of safe blood transfusions in Walikale hospital. These activities were extended in September 2012 with support provided to six basic health centres (BHC) from the Ministry of Health (MoH) in the area. According to the MSF outpatient department (OPD) data in the Walikale project, between June 2012 and May 2013, 84,005 consultations were conducted with an average of 7000 consultations per month. Proportionally, confirmed malaria represented the highest morbidity for consultation (32.8% of monthly consultations) followed by acute upper respiratory tract infections and intestinal parasites, with 14.1% and 10.5% of the total consultations (all ages) respectively.

To better understand the impact of the recent violence and population movements in Walikale and surrounding areas on the humanitarian and healthcare needs in the population, MSF conducted a retrospective household mortality in April 2013. The principle objective of the survey was to estimate the crude mortality rate (CMR) and under-five mortality rate (U5MR) in the population living in the Walikale catchment area. The secondary objectives were to determine the frequency and reasons for displacement, to assess the populations' access to health care, to determine the main reported reasons for death, to measure the number and types of violence experienced by the population and to determine ownership of basic non-food items (NFIs). The results from the survey were used immediately to adapt the programme needs and improve health services offered to the host and displaced populations.

## Methods

### Study area

The study was conducted in the MSF catchment area for the Walikale project, including most of the Walikale Health Zone, and parts of the Kibua and Itebero Health Zones. Villages that our interview team could not reach and return from within one day (for security reasons) were excluded from the sampling frame.

### Sample size

The sample size was calculated using ENA for SMART software [9] assuming that the CMR was 0.7 per 10,000 per day for a recall period of 325 days with a precision of 0.3, a design effect of 3 and a non-response rate of 10%. This CMR estimate was based on the results from previous retrospective mortality surveys conducted in North Kivu in 2009 [7].

The sample size calculated included 613 households with 3065 persons assuming a mean household size of five people.

### Study design

Our sampling frame contained 147 villages in the Walikale project catchment area representative of a population of 160,305 persons based on the 2011 MoH census data adjusted to 2013 with a growth factor. As systematic random sampling was not feasible in this context, we conducted two-stage cluster sampling as described elsewhere [10]. For the first stage, we systematically sampled 31 clusters from the sampling frame proportionate to the respective population size of each village (PPS). In the second stage, 21 households per cluster were sampled at random using the modified EPI method [10]. Interviewers were instructed to return to empty houses later in the day when the inhabitants were present. If the household was determined to be uninhabited at the time of the interview, the next closest household was chosen.

### Definitions

A household was defined as a group of people who were under the responsibility of one person or head of household, regularly sleeping under the same roof and eating together (in a single dwelling). All household members were included, no matter the age of the household member or the relation with the other members.

The head of household had to meet the following criteria: adult household member (15 years or more), who could give accurate information on all demographic and mortality issues in his/her household and who had also lived in the household regularly for the entire recall period, and was present at the time of the survey.

### Data collection

The household interviews were based on a structured questionnaire that was divided in two sections and answered by the head of the household. The first part collected information on the origin of the household (returned, displaced, permanent), the condition of their goods and house in their place of origin, access to NFIs (bednets etc.), and access to health-care. The access to health care was evaluated by asking about the last person that had been sick in the last two weeks prior to the survey. The second part asked the age and sex of individual household members and dates of arrival/departure/births and deaths during the recall period. For all deaths, we asked about the reported cause of death. We also asked the head of the household to report on the number and types of episodes of violence (by armed person, with weapon, sexual violence) experienced by individual household members during the recall period. The questionnaires were translated and back-translated to ensure consistency from English, to French and Swahili. Interviews were conducted in Swahili.

Sixteen locally recruited skilled persons were trained as interviewers for two days on the methodology of the survey and how to complete the questionnaire. A third day of training was used to pilot the questionnaire in the field, and correct errors in interviewing techniques from the interview team.

### Recall period

The recall date was the 30th of June, 2012, which is the anniversary of independence of DRC and therefore easily remembered by the whole population (325 days from the date of the survey). Also, this date was two weeks prior to the intense fighting that occurred in Walikale town and in the surrounding area in July 2012. A secondary recall date was also included in relation to the occurrence of disease in the members of the household in the 14 days preceding the date of the survey being carried out.

### Data entry and analysis

Data was entered into EpiData 3.0 software (The EpiData Association, Odense, Denmark) by the field epidemiologist and a data encoder. Ten percent of all questionnaires were randomly selected to check the quality of the data entry. Data analysis was conducted using ENA for SMART [9] and STATA 12 (StataCorp, College Station, TX, USA). To determine statistical differences between proportions the Mantel-Haenszel Chi Square was calculated with its respective p-value.

We did not collect precise dates of arrival, departure and births of household members in this survey. Therefore, the denominator used for the calculation of the mortality rates corresponded to the mid-recall-period population size. This method has been described elsewhere [11] and assumes that the denominator is the total population at the end of the recall period minus half of persons joining the sample during the recall period (newborns and joining household members) plus half of persons leaving the sample during the recall period (because of death or absenteeism).

All indicators (i.e. sex and age of the survey population) were calculated as proportions with 95% confidence intervals (CI95%) in STATA to incorporate the cluster design effect. Estimates of actual design (cluster) effect (DEFF) were also calculated for the mortality rates calculated using ENA software.

### Ethical considerations

The heads of the villages were informed about the survey, in writing, at least two days prior to the arrival of the interviewers. They were also visited on the day of the survey to ensure they were aware of the team's presence and planned activities. The heads of the villages as well as selected households were informed they could decline the participation of their village/household without any consequences or penalty. In selected households the purpose of the survey was explained to the head of the household in Swahili and written consent was obtained before conducting the interviews. When the interviewees were not able to sign, we asked for their fingerprint. All collected data was anonymous.

This survey was approved by the MSF-OCA Ethical Review Board on 4 February, 2013 and it subscribes to the ethical principles outlined in the Declaration of Helsinki [12]. The protocol and questionnaires were shared with the MoH of DRC, prior to implementation of the study and approval was obtained for the implementation of the survey.

## Results

A total of 31 clusters of 21 households were surveyed between 21 May and 3 June, 2013. No selected village declined to participate in the survey. A total of 651 households containing 4,157 living members were interviewed. Only five households (0.8%) refused to participate in this survey, four of them because they were leaving soon or they were very busy, and one stated that they were not interested in any interview. The mean household size of all households at the time of the interview was 6.4 people (CI95%: 6.0–6.8).

### Demographic information and household status

**Figure 2:**
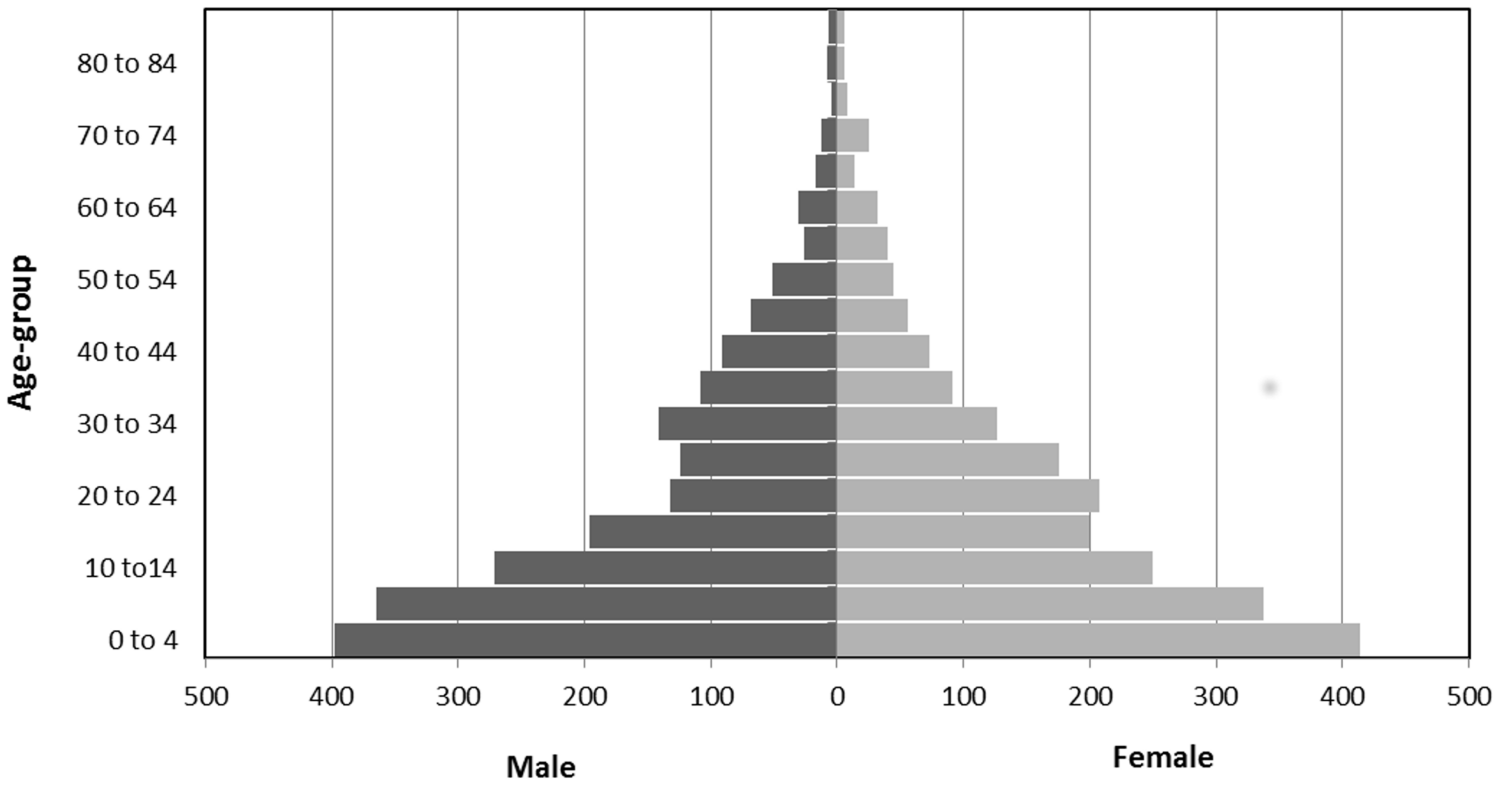
Population pyramid, retrospective mortality survey, Walikale, North Kivu, DRC, June 2013 (N = 3,983).

The median and mean age of the surveyed population was 15 years and 20 years respectively (range: 0–94). Children under 5 years of age made up 19.5% (n = 812, CI95%: 18.1–21.0) of the population (N = 4157). The male/female ratio was 0.97 (2052 vs. 2105). A population pyramid was constructed using the studied individuals alive at the time of the survey (n = 3983) (Figure 2). In the age group of 20 to 29 years, the male to female ratio was 0.65 (247 vs.379). In all other age groups, this ratio was closer to 1.0.

The majority of interviewed households (n = 267, 41.8%, CI95%: 29.9–52.1) classified themselves as returned (families that had been displaced in the past, then came back and at the time of the interview they were living in their village of origin), followed by permanent residents (those who were not displaced) (n = 201, 30.9%, CI95%: 20.5–41.2) and displaced (families displaced and living in a different village at the time of the interviews) (n = 181, 27.8%, CI95%: 19.8–35.8). The reason for displacement during the recall period was available for 403 (90.0%, CI95%: 86.9–92.5) of the displaced and returned households. Similar proportions of households reported that the reason for displacement was a direct attack (n = 184, 45.7%, CI95%: 40.8–50.5) and insecurity (n = 165, 40.9%, CI95%: 36.2–45.8). Fifty-four households (13.4%, CI95%: 10.3–17.0) reported that both direct attack and insecurity had been the reason of their displacement.

### Retrospective mortality

During the recall period of 325 days, 134 births and 173 deaths were reported in the survey population. Of the deaths, 44 (25.4%, CI95%: 19.4–32.3) were in children under five years of age. Among the total number of deaths reported for who the information was available, 92 (53.2%, CI95%: 45.7–60.5) occurred among males. Also, 1,988 individuals (47.8%, CI95%: 46.3–49.3) were reported to have both left and arrived (or the other way around) in the household during the recall period. Household heads reported 168 individual departures (4.0%, CI95%: 3.5–4.7) from their household and 951 individual arrivals (22.9%, CI95%: 21.6–24.2) during the recall period. The calculated CMR for the surveyed population was of 1.4 deaths/10,000 population/day (CI95%:1.2–1.7, DEFF = 2.9) and an U5MR of 1.9 deaths/10,000 population/day (CI95%: 1.3–2.5, DEFF = 1.0).

### Reported causes of death

Overall, the most frequently reported cause of death was fever/malaria (n = 59, 34.1%, CI95%: 25.4–42.9). This was also the main reported cause of death in both the <5 years and ≥5 years age group (Table 1). In children under 5 years, death from diarrhea or measles was the second and third most commonly reported cause of death respectively. In persons older than 5 years of age, similar proportions of causes of death were reported for respiratory problems, intentional violence and hernia (Table 1 and footnote). Eleven deaths (8.5%, CI95%: 3.0–14.0) were reported in women due to reasons associated with pregnancy. Out of all reported deaths, 14 were in children under 12 months of age (8.1%, CI95%: 4.7–12.9). For nine of these deaths (64.3%) the reported cause of death was fever/malaria (Table 1).

**Table 1:** Reported causes of death during recall period, by age group and overall, Walikale, North Kivu, DRC, June 2013.

	<5 years		≥5 years		Overall	
	*n*	*% (CI95%)*	*n*	*% (CI95%)*	*n*	*% (CI95%)*
Fever/malaria	25	56.8 (38.4–75.3)	34	26.4 (16.0–36.7)	59	34.1 (25.4–42.9)
Diarrhea	8	18.2 (5.9–30.5)	20	15.5 (8.4–22.7)	28	16.2 (8.8–23.6)
Measles	5	11.4 (4.3–23.4)	0	–	5	2.9 (1.1–6.3)
Respiratory problem	2	4.5 (−2.4–11.5)	11	8.5 (4.7–12.4)	13	7.5 (3.7–11.3)
Intentional violence	1	2.3 (−2.5–7.0)	12	9.3 (4.6–14.0)	13	7.5 (3.9–11.1)
Accidental violence	1	2.3 (−2.5–7.0)	9	7.0 (1.0–13.0)	10	5.8 (1.2–10.3)
Pregnancy*	*na*	*na*	11	8.5 (3.0–14.0)	11	6.4 (2.4–10.3)
*Hernia* **	*na*	*na*	8	6.2 (2.9–11.4)	8	4.6 (2.2–8.6)
Other	2	4.5 (0.8–14.2)	23	17.8 (11.9–25.2)	25	14.5 (9.8–20.3)
Missing value	0	–	1	0.8 (−0.8–2.4)	1	0.6 (0.6–1.8)
TOTAL	44	100	129	100	173	100

* includes death in mother up to one month after birth of child.

** the term “hernia” is often used in this part of Congo to illustrate symptoms of other diseases or it is thought to provoke other diseases, as it was not clinically assessed it should not be interpreted as the medical definition of this term.

There was no significant difference between the proportion of deaths reported by household status (Table 2). In all three categories of household status, fever/malaria was the main reported cause of death (more than 30%). Permanent households reported no deaths in the recall period from intentional violence, whereas 14% of deaths in displaced households were reportedly from this cause (Table 2).

**Table 2:** Reported causes of death by household status, Walikale, North Kivu, DRC, June 2013.

	Displaced		Returned		Permanent	
	*n*	*% (CI95%)*	*n*	*% (CI95%)*	*n*	*% (CI95%)*
Fever/malaria	15	30.0 (5.1–24.9)	33	38.8 (23.0–43.0)	11	31.4 (0.6–21.4)
Diarrhea	8	16.0 (0.5–15.5)	11	12.9 (4.4–17.7)	9	25.7 (−0.5–18.5)
Respiratory problem	1	2.0 (−1.8–3.8)	9	10.6 (2.9–15.1)	2	5.7 (−2.6–6.6)
Intentional violence	7	14 (−0.1–14.1)	5	5.9 (0.4–9.6)	0	0
Accidental violence	3	6 (−1.7–7.7)	5	5.9 (0.4–9.6)	2	5.7 (−2.6–6.6)
Pregnancy*	5	10 (−1.0–11.0)	5	5.9 (0.4–9.6)	1	2.9 (−2.3–4.3)
Other	11	22.0 (2.3–19.7)	17	20.0 (9.0–25.0)	10	28.6 (0.1–19.9)
TOTAL	50	29.2 (42.5–57.5)	85	49.7 (79.7–90.4)	35	20.4 (27.9–42.2)

* includes death in mother up to one month after birth of child.

### Household ownership of basic non-food items (NFI)

The majority of households (n = 449, 69.0%, CI95%: 61.7–76.3) owned at least one jerry can of 10 Liters, but only 22.6% (CI95%: 18.0–27.2) of those jerry cans could close properly. More than half of surveyed households reported ownership of a mattress and cooking utensils, 59.3% (CI95%: 52.7–65.9) and 74.7% (CI95%: 68.5–80.8) respectively. Blankets were owned by 23.7% (CI95%: 14.9–32.4) of households. Soap was present and observed in only 30.1% (CI95%: 24.3–35.9%) of the households interviewed. At least one bed net was owned by 56.5% of responding households (n = 368, CI95%: 49.7–63.3). Among the 341 households which provided detailed information on bed net ownership, an average of 1.6 mosquito nets was available per household. Almost half of households (n = 308, 47.3%, CI95%: 38.7–56.0) had a piece of land which they could use for cultivation and 28.4% (CI95%: 23.2–33.6) of households had a partial piece of land. However, only 30.4% (CI95%: 24.3–36.5) of all households reported owning agricultural tools. When comparing ownership of all mentioned items in displaced households compared to returned and permanent households combined, displaced households had a significantly lower ownership of NFIs (31.7%, CI95%: 25.8–38.2) compared to that in returned and permanent households (55.6%, CI95%: 51.4–59.9) (Chi-square Mantel-Haenszal = 29.77, p<0.001).

### Access to health care

In the two weeks preceding the survey, 89.0% of the interviewed households (n = 580. 95% CI95%: 85.4–92.8) reported they had had at least one person sick in their household. In 40.3% (n = 234, CI95%:40.3–44.4) of these cases, the person had been under 5 years of age. The two most commonly reported disease among all persons reported to be sick in the previous two weeks was fever/malaria and diarrhea (Table 3). Pregnancy and childbirth related complaints were the third most common reason for illness.

**Table 3:** Reported diseases for persons seeking care two weeks prior to the survey by age group and overall, Walikale, North Kivu, DRC, June 2013.

	<5 years		≥5 years		Overall	
	n	% (CI95%)	n	% (CI95%)	n	% (CI95%)
Fever/malaria	152	65.0 (57.6–72.4)	175	50.6 (44.4–56.7)	327	56.4 (52.2–60.5)
Diarrhea	63	26.9 (20.5–33.4)	41	11.8 (8.1–15.6)	104	17.9 (14.7–21.1)
Respiratory infection	9	3.8 (0.8–6.8)	14	4.0 (2.4–5.7)	23	4.0 (2.4–5.5)
Accident	0	0	5	1.4 (0.2–2.7)	5	0.9 (0.1–1.6)
Pregnancy related	na	na	44	12.7 (8.4–17.1)	44	7.6 (5.2–10.0)
Childbirth related	na	na	1	0.3 (−0.3–0.9)	1	0.2 (−0.2–0.5)
Others	10	4.3 (1.4–7.2)	66	19.1 (13.3–24.9)	76	13.1 (9.0–17.2)
TOTAL	234	40.3 (36.4–44.4)	346	59.7 (55.6–63.6)	580	

Among all the households reporting somebody sick in the previous two weeks, 63.8% (n = 370, CI95%: 57.1–70.5) sought care in a health structure. Among care seekers, 66.4% (n = 245, CI95%:53.1–79.7) had to pay for the consultation. The main reason for not seeking care in a health structure was the lack of money (63.8%, n = 134, CI95%: 52.2–75.4). Other common reasons for not seeking care included: having purchased medication (n = 84, 40.0%, CI95%: 52.2–75.4), use of traditional medicine (n = 21, 10.0%, CI95%: 5.7–14.3), and the person was not ‘very’ sick (n = 15, 7.1, CI95%: 2.4–11.9). The sense of a lack of security to seek out healthcare was mentioned by only one respondent (0.5%, CI95%: 0.5–1.4).

### Episodes of violence and theft/destruction of property

Around (6.4%, n = 265, CI95%: 5.1–7.6) of the total study population reported having been the victim of one or more episodes of violence during the recall period. The mean number of violent incidents per person (among the 265 individuals that suffered from violence) was 2.3 episodes and the majority of violent incidents were reported by males (n = 178, 67.1%, CI95%: 59.4–74.9). The most frequently reported type of violence was having been beaten (n = 135, 50.9%, CI95%: 43.3–58.6). In 26.8% (n = 71, CI95%: 18.0–35.6) of those violent episodes, the reported outcome was having been detained or kidnapped. Sexual violence was the third most commonly reported cause (n = 34, 12.8%, CI95%: 7.7–17.9); only one was reported by a male member of the study population. Sixteen persons (6.0%, CI95%: 3.0–9.1) were reported victims of gun shots and six persons victims of a bladed weapon (2.3%, CI95%: 0.5–4.1). Most of the individuals who reported violence (81.9%, n = 217, CI95%: 73.7–90), confirmed the perpetrator wore a uniform. Episodes of violence were most common between the ages of 15 and 49 years (n = 113, 42.6%, CI95%: 37.7–47.6) and in men (n = 178, 67.1%, CI95%: 59.4–74.9).

Out of all households, 513 (78.9%, CI95%: 71.7–85.9) reported having had household goods and/or livestock/cattle destroyed or stolen during the recall period. Twenty-five percent (n = 166, CI95%: 19.7–31.3) of the interviewed household reported the status of his house as partially destroyed after the violent incidents in July 2012. In 63 households (9.7%, CI95%: 5.0–14.3) the houses were reported to be completely destroyed.

## Discussion

This survey in Walikale illustrates the effects of the ongoing conflict, violence and displacement on the population in this part of DRC, but also the continued chronic burden of infectious diseases at the community level. The gravity of the population's situation is highlighted by a CMR that is well above the emergency threshold and an U5MR that approaches it [11] for the recall period between July 2012 May 2013. The proportion of displaced households (from direct attack and insecurity) is high, access to healthcare is limited (mostly due to economic constraints) and low levels of NFI ownership in households highlight the precarious living conditions of the population. While violent incidents are common (7.5% of all reported deaths), the main reported cause of mortality and reason to seek health care in this population is from preventable and/or treatable infectious diseases, namely malaria and diarrhea. In contrast, previous mortality surveys from 2009 reported mortality rates below the emergency threshold with higher proportions of death due to violent incidents (13.3%, 39.7% and 35.9%) [7].

The average household size in the surveyed population was slightly larger (6.6 persons) than that used to estimate the sample size (5 persons) and may reflect the consolidation of displaced persons with other families in the area at the time of the survey. A similar average household size was estimated in Masisi, North Kivu, in 2009 by Alberti et al. [7]. The proportion of children under the age of five is similar to that in other low-resource settings, between 15–20% of the population [13].

The impact of the conflict on the population can also be seen in that the majority of violent incidents were reported in young adult men and that the proportion of males in the 20–29 years age group is visibly absent in the population pyramid. The reasons for this can be speculated to be because of conscription into armed groups, kidnapping or death from direct violence as a result of the conflict. The relation between the ongoing conflict and the reported violent episodes is highlighted by the high frequency of perpetrators that reportedly wore a uniform. Sexual violence was the third most commonly reported nature of violence. A study in the Eastern Region of the DRC found a sexual violence incidence of 39.7% in 2010 [14]. Consequently the sexual violence incidents in our survey (12.8%) are probably underreported due to the sensitive nature of this topic and difficulty in asking questions related to it during a household survey.

This study found a higher proportion of households that reported a sick household member in the two weeks preceding the survey, than that estimated by Alberti et al. in 2009 (70% for all the studied sites) [11]. The most commonly reported cause of morbidity and mortality in all age groups (and regardless of household status) was fever (malaria), with diarrhea, measles and pregnancy related problems also being clear concerns. These reflect the health status of populations in other sub-Saharan African countries where 55% of deaths in children are caused by malaria, pneumonia and diarrhea [15]. The finding is also confirmed by the data from the outpatient departments in Walikale where the highest reported morbidity between October 2012 and June 2013 was confirmed malaria, representing 25% of all consultations in this period. Crucially, the ownership of an average of 1.6 bednets for an average household size of 6.6 persons is critically low when compared to the established standard of two persons per net [16]. This illustrates the absence of basic preventative materials for malaria and other vector-borne diseases in this part of DRC.

The survey has been limited by collecting the causes of morbidity and mortality from a single household member and not using formal verbal autopsy methodologies therefore potentially leading to an over or under estimation of the true burden of disease in the surveyed population. Certain causes of death, such as malaria and respiratory diseases, can be difficult to diagnose and therefore the understanding of causation is limited [13]. Also, the self-reporting of disease is more accurate for children, specifically for neonatal tetanus and measles where the symptoms are very particular [11]. The high number of reported persons having died of ‘hernia’ in this survey is a clear example of the limits of self-reporting causes of deaths, where the word refers to diseases affecting the abdominal area. Also, household repondents reported on ‘fever’ which was interpreted as a proxy for malaria. However, fever is a symptom of various other diseases, including acute respiratory infection and parasitic infections, and therefore the burden of these diseases was probably underreported in this survey.

In contexts like North Kivu, there needs to be a constant balance between logistical and security constraints and the need for accurate data. The sample size of 31 clusters was sufficient to measure the main outcome of mortality in the target population with enough precision; however, a stratified analysis on geographical sub-area was not possible. Also, several very distant villages were excluded from the initial sampling frame. This has probably led to an underestimation of the CMR and U5MR as these villages are even further removed from formal healthcare and they are more prone to violence and conflict.

The recall period was at the higher limit of acceptable recall periods (325 days), which could have led to significant recall bias from the respondents [11]. Even though the long recall period provides a higher precision in the mortality rate estimation people will have more difficulty remembering events, consequently mis-reporting their occurrence (episodes of violence etc.). The recall bias was minimized by choosing a recall date that is well-known and remembered in this part of DRC. Around this recall date in 2012 there was significant fighting in Walikale (second and third week of July) which was well remembered by most respondents. With regards to NFI ownership and household size, respondents might have misinterpreted these questions as part of a population registration exercise and might therefore have inflated the number of individuals in the household and underreported their NFI ownership. Reporting bias was minimized by clarifying the importance of accurate information with the respondents and by training the interviewers to visually verify the NFI items. The only households interviewed were obviously those for whom at least one member was alive (survival bias) [11], therefore the households whose members died or whose orphans had joined other households were not registered and consequently the mortality rates could be underestimated.

Another important limitation is that household respondents were not asked to give precise dates of the arrival, departure and birth of household members. Providing such detailed information would be difficult considering the long recall period. However, the lack of the total person-days contributions for each individual in the survey has limited the calculation of a precise CMR and U5MR, instead relying on an estimate of the total denominator as used in previous surveys [11]. Even so, the current mortality rates are probably the most conservative considering that more accurate person-day calculations would have reduced the overall denominator even further, thereby raising the mortality rate.

This survey has demonstrated that in areas of conflict, such as Walikale, even though violence remains an important reason for concern, common preventable and easily-treatable diseases such as malaria and diarrhea are of great concern also. As a direct result of the findings of this survey in Walikale, MSF has expanded its community based activities in order to have closer contact with the population to detect, diagnose and treat cases of disease earlier, reducing morbidity and mortality. These activities have included reinforcing the community health network which exists in most villages in North Kivu by strengthening surveillance activities and speeding up referral options for severely sick persons. It has also included an increased number of mobile clinics to allow for a decentralized access to free health care, including diagnosis and treatment of malaria, diarrhea and respiratory infections. These strengthened medical and surveillance activities will provide more concrete insights of the actual diseases that burden these communities (be they neglected tropical diseases or others). A new retrospective mortality survey will be repeated in 12 months following this one to understand whether the MSF activities have managed to impact in a positive way on the mortality and health status of this vulnerable population in Walikale.

## Supporting Information

Checklist S1 STROBE checklist.
(DOC)

Text S1 Household questionnaire, Walikale Retrospective Mortality Survey, May–June, 2013.
(DOC)

Text S2 Individual questionnaire, Walikale Retrospective Mortality Survey, May–June, 2013.
(DOC)
