# Supplementary material for: Mortality Rates above Emergency Threshold in Population Affected by Conflict in North Kivu, Democratic Republic of Congo, July 2012–April 2013
Source: PLoS Negl Trop Dis. 2014 Sep 18;8(9):e3181. doi: 10.1371/journal.pntd.0003181 (PMC4169374; doi:10.1371/journal.pntd.0003181)
Supplement: Checklist S1 — STROBE checklist. (DOC) [file pntd.0003181.s001.doc]

STROBE Statement—Checklist of items that should be included in reports of ***cross-sectional studies***

*The checklist items have been addressed by the authors in “italics” under each checklist item.*

|  | Item No | Recommendation |
| --- | --- | --- |
| **Title and abstract** | 1 | (*a*) Indicate the study’s design with a commonly used term in the title or the abstract |
|  |  | (*b*) Provide in the abstract an informative and balanced summary of what was done and what was found  *This is done in both the title and abstract* |
| Introduction | | |
| Background/rationale | 2 | Explain the scientific background and rationale for the investigation being reported  *Described in the first two paragraphs of the introduction* |
| Objectives | 3 | State specific objectives, including any prespecified hypotheses  *Described in the last paragraph of the introduction* |
| Methods | | |
| Study design | 4 | Present key elements of study design early in the paper  *Sample size, sample populations and study design are the first three paragraphs of the methods* |
| Setting | 5 | Describe the setting, locations, and relevant dates, including periods of recruitment, exposure, follow-up, and data collection  *This is all address in the respective sections of the Methods.* |
| Participants | 6 | (*a*) Give the eligibility criteria, and the sources and methods of selection of participants  *Section on definitions highlights who was eligible to be interviewed.* |
| Variables | 7 | Clearly define all outcomes, exposures, predictors, potential confounders, and effect modifiers. Give diagnostic criteria, if applicable  *Included in the section that describes the questionnaire that was used* |
| Data sources/ measurement | 8* | For each variable of interest, give sources of data and details of methods of assessment (measurement). Describe comparability of assessment methods if there is more than one group  *No comparison group was available, all other aspects defined in data collection and data analysis part of the Methods* |
| Bias | 9 | Describe any efforts to address potential sources of bias  *Cluster allocation proportional to population size and household random selection were the main methods used to reduce bias. These are described.* |
| Study size | 10 | Explain how the study size was arrived at  *Separate section on sample size included.* |
| Quantitative variables | 11 | Explain how quantitative variables were handled in the analyses. If applicable, describe which groupings were chosen and why  *Separate section on data analysis addresses this.* |
| Statistical methods | 12 | (*a*) Describe all statistical methods, including those used to control for confounding  *This is covered in the data analysis section.* |
|  |  | (*b*) Describe any methods used to examine subgroups and interactions  *We did not do further stratification as the sample size did not allow for this.* |
|  |  | (*c*) Explain how missing data were addressed  *There was no missing data as such as the target of households in the survey was achieved.* |
|  |  | (*d*) If applicable, describe analytical methods taking account of sampling strategy  *Included in the data analysis section.* |
|  |  | (*e*) Describe any sensitivity analyses  *Not applicable.* |
| Results | | |
| Participants | 13* | (a) Report numbers of individuals at each stage of study—eg numbers potentially eligible, examined for eligibility, confirmed eligible, included in the study, completing follow-up, and analysed  *Covered in the first paragraph of the results section.* |
|  |  | (b) Give reasons for non-participation at each stage  *Number of houses that refused to participate are indicated.* |
|  |  | (c) Consider use of a flow diagram  *Not considered to be of added value for this study applicable* |
| Descriptive data | 14* | (a) Give characteristics of study participants (eg demographic, clinical, social) and information on exposures and potential confounders  *Included throughout the results section by specific area of interest.* |
|  |  | (b) Indicate number of participants with missing data for each variable of interest🡪 *The only question for which data was missing (due to inconsistency in asking for the first half of the survey), relates to the ownership of bednets. It is stated clearly that this information was only available for 341 out of the 651 households interviewed.* |
| Outcome data | 15* | Report numbers of outcome events or summary measures  *Outcomes are clearly communicated in each of the respective section in the results.* |
| Main results | 16 | (*a*) Give unadjusted estimates and, if applicable, confounder-adjusted estimates and their precision (eg, 95% confidence interval). Make clear which confounders were adjusted for and why they were included  *No multivariate analysis was done, therefore not applicable* |
|  |  | (*b*) Report category boundaries when continuous variables were categorized  *Where relevant, this was included.* |
|  |  | (*c*) If relevant, consider translating estimates of relative risk into absolute risk for a meaningful time period  *Not applicable.* |
| Other analyses | 17 | Report other analyses done—eg analyses of subgroups and interactions, and sensitivity analyses  *Not applicable* |
| Discussion | | |
| Key results | 18 | Summarise key results with reference to study objectives  *First paragraph of the discussion* |
| Limitations | 19 | Discuss limitations of the study, taking into account sources of potential bias or imprecision. Discuss both direction and magnitude of any potential bias  *This is addressed in several paragraphs of the discussion.* |
| Interpretation | 20 | Give a cautious overall interpretation of results considering objectives, limitations, multiplicity of analyses, results from similar studies, and other relevant evidence  *Included in the discussion.* |
| Generalisability | 21 | Discuss the generalisability (external validity) of the study results  *First sentence of the last paragraph of the discussion.* |
| Other information | | |
| Funding | 22 | Give the source of funding and the role of the funders for the present study and, if applicable, for the original study on which the present article is based  *No separate source of funding was sought for this survey. It was conducted within the operational budget of the Walikale project in DRC. We therefore have not included a section on funding.* |

*Give information separately for exposed and unexposed groups.

**Note:** An Explanation and Elaboration article discusses each checklist item and gives methodological background and published examples of transparent reporting. The STROBE checklist is best used in conjunction with this article (freely available on the Web sites of PLoS Medicine at http://www.plosmedicine.org/, Annals of Internal Medicine at http://www.annals.org/, and Epidemiology at http://www.epidem.com/). Information on the STROBE Initiative is available at www.strobe-statement.org.
