# Supplementary material for: Mortality Rates above Emergency Threshold in Population Affected by Conflict in North Kivu, Democratic Republic of Congo, July 2012–April 2013
Source: PLoS Negl Trop Dis. 2014 Sep 18;8(9):e3181. doi: 10.1371/journal.pntd.0003181 (PMC4169374; doi:10.1371/journal.pntd.0003181)
Supplement: Text S1 — Household questionnaire, Walikale Retrospective Mortality Survey, May–June, 2013. (DOC) [file pntd.0003181.s002.doc]

**Questionnaire, retrospective mortality survey, Household level, Walikale, DRC 2013**

| **Village name:** |  | **Cluster #:** |  | **Date:** |  |
| --- | --- | --- | --- | --- | --- |
| **Team #:** |  |  | | **Time start:** |  |
| **HH #:** |  | **Family#:** |  | **Time end:** |  |

| **Household situation** |
| --- |

1. **Village of origin of the family/household**:________________________________________________
2. **Situation of family**:
3. Displaced
4. Returned; *If returned or permanent, skip to question 4 otherwise go to question 3*
5. Permanent
6. **If displaced, provide reason and number of displacements in the table below**:

| Reason | Number of times during recall period |
| --- | --- |
| Attacked |  |
| In security |  |
| Ordered to leave |  |
| Other, specify ____________________ |  |

| 1. **Damage/stolen to household goods and items**    1. Yes *–*    2. No - | 1. **Which household items were stolen?**    1. Household goods    2. Cattle/livestock    3. Household goods and cattle/livestock |
| --- | --- |
| 1. **What happened to your home :** 2. Destroyed completely 3. Destroyed partially 4. Occupied (by someone else) 5. Abandoned 6. Don’t know |  |

| **NFI possession** |
| --- |

7. **Possession of non-food items in the household (please circle the correct responses):**

1. Jerrycan 10 liter capacity (minimum): Yes No
2. If yes, does it close properly: Yes No
3. How many blankets do you have that are in good condition? (NB: this means that there are less than 10% of holes in the total surface): __________
4. Do you have mosquito nets? Yes No
5. If yes, how many mosquito nets do you own? _______________
6. Cooking items (at least one cooking pot and one large cooking spoon): Yes No
7. Agricultural tool (minimum 1 hoe/shovel) Yes No
8. Access to land for cultivation Yes No Partially

| **Access to health care** |
| --- |

1. **Has someone in your family/household been seek in the last two weeks? Yes No**

*If yes, continue with question 9. If no, continue to the individual questions*

1. **Who was the last person to be sick in your family in the last two weeks?**

_____________________________________________________________________________

| 1. **Sex**: _____ (F/M) | 1. **Age**: _____ years ou _____ months |
| --- | --- |
| 1. **Which disease did the person have?**    1. Diarrhea    2. Respiratory infection    3. Fever/malaria    4. Pregnancy related    5. During or after the birth of a child (less than 1 month old)    6. Accident    7. Violence    8. Doesn’t know    9. Other disease (specify) | 1. **Did this person receive health care?** Yes No   *If yes, go to question 14, if No go to question 15* |
| 1. **If yes, specify which health facility they visited :**    1. Hospital    2. Health post    3. Health centre    4. Private healthcare centre    5. Traditional medical person    6. Auto medicated | 1. **If no, why did they not receive any health care?**    1. They weren’t sick enough    2. The health post/centre was too far    3. We found medication in the market or at the pharmacy    4. We used traditional medicine    5. We didn’t have the financial means to go    6. We don’t trust the healthcare services offered    7. There were secruirty problems    8. We didn’t have time to go    9. The person was refused at the healthcare facility    10. Another reason (specify): __________________________________ |
| 1. **How many days after the person was sick did you seek healthcare?**    1. Same day    2. 2-7 days after    3. 8-14 days after | 1. **Which health facility did you visit?**    1. MSF    2. Ministry of Health    3. NGO    4. None    5. Other    6. Don’t know |
| 1. **Did you pay for the consultation?** Yes No | 1. **If no, why?**    1. Not enough financial resources    2. Security problem    3. Other (specify) |
