# Supplementary material for: Mortality Rates above Emergency Threshold in Population Affected by Conflict in North Kivu, Democratic Republic of Congo, July 2012–April 2013
Source: PLoS Negl Trop Dis. 2014 Sep 18;8(9):e3181. doi: 10.1371/journal.pntd.0003181 (PMC4169374; doi:10.1371/journal.pntd.0003181)
Supplement: Text S2 — Individual questionnaire, Walikale Retrospective Mortality Survey, May–June, 2013. (DOC) [file pntd.0003181.s003.doc]

**Team number: …………………………… Date : ………… /……………/……………..**

**Cluster number :………………… Household number :…………………. Family number :…………………….**
